# Computation of a probabilistic uranium concentration map of Norway: A digital expert elicitation approach employing random forests and artificial neural networks

**DOI:** 10.1016/j.heliyon.2023.e21791

**Published:** 2023-10-31

**Authors:** Hendrik Paasche, Ying Wang, Vikas Chand Baranwal, Marco Brönner

**Affiliations:** a UFZ – Helmholtz Centre for Environmental Research GmbH, Department Monitoring and Exploration Technologies, Permoserstr. 15, 04318 Leipzig, Germany; b Geological Survey of Norway (NGU), Leiv Eirikssons vei 39, 7040 Trondheim, Norway

**Keywords:** Expert elicitation, Uncertainty quantification, Monte Carlo, Model uncertainty, Uranium concentration, Norway, Non-linear regression, Geophysics, Radiometry

## Abstract

We compute the first probabilistic uranium concentration map of Norway. Such a map can support mineral exploration, geochemical mapping, or the assessment of the health risk to the human population. We employ multiple non-linear regression to fill the information gaps in sparse airborne and ground-borne uranium data sets. We mimic an expert elicitation by employing Random Forests and Multi-layer Perceptrons as digital agents equally qualified to find regression models. In addition to the regression, we use supervised classification to produce conservative and alarmistic classified maps outlining regions with different potential for the local occurrence of uranium concentration extremes. Embedding the introduced digital expert elicitation in a Monte Carlo approach we compute an ensemble of plausible uranium concentrations maps of Norway discretely quantifying the uncertainty resulting from the choice of the regression algorithm and the chosen parametrization of the used regression algorithms. We introduce digitated glyphs to visually integrate all computed maps and their associated uncertainties in a loss-free manner to fully communicate our probabilistic results to map perceivers. A strong correlation between mapped geology and uranium concentration is found, which could be used to optimize future sparse uranium concentration sampling to lower extrapolation components in future map updates.

## 1 Highlights

• Uncertainty quantification by means of digital agents in an expert elicitation.
• Computation of a probabilistic uranium concentration map of Norway.
• Full map uncertainty communication to the perceiver by means of glyphs.
• Mapped geology may guide future U data acquisition to reduce extrapolation.

## Graphical abstract

**Figure:**
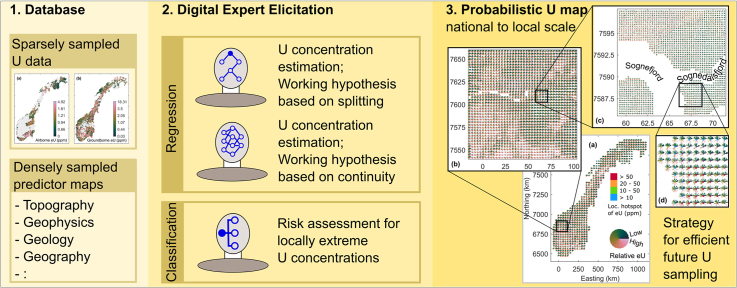

## 1 Introduction

Knowledge of the natural ionizing background radiation is important information essentially required for mineral exploration, geochemical and geological mapping, and assessment of the health risk to the human population as well as for the development of appropriate protection strategies where required [1]. Particularly in Norway, where the shielding soil cover of bedrocks containing radioactive elements, such as Thorium, Uranium, or Potassium, is mostly thin or absent, long-lasting exposure of the human and life stock populations to high geogenic radiation dose rates or radon gas concentrations is a long-lasting concern [2].

Many studies, predominantly conducted on local to regional scale, have been directed towards radon risk mapping in Norway and elsewhere. Some authors employ measurements of indoor radon concentrations, despite its dependency on anthropogenic aspects such as building materials or indoor air exchange [[3], [4], [5], [6], [7]]. Other authors focus on the geogenic radon potential measuring the radon concentration in natural soil gas [[8], [9], [10], [11]]. In addition to a relation of radon gas concentration to mapped geology [4], many authors report a positive correlation of radon gas concentration and uranium concentration in the near surface ground suggesting to use measurements of natural gamma radiation and uranium concentrations in particular as proxy to judge the radon risk [[2], [6], [9], [12], [13], [14]],. Measurements of natural gamma radiation allow also for the estimation of a total radiation dose rate exposure [[15], [16], [17]]. To compute a countrywide dose rate exposure map, maps of Thorium, Potassium, and Uranium concentrations are required. Frequently, the results of airborne radiometric surveys, dose rate maps, radon hazard maps, or radiation risk maps are presented as deterministic results without quantification of uncertainties associated to the considered data and data processing methods.

While some countries have completed or almost completed national airborne gamma radiation mapping surveys (e.g., Ireland and Northern Ireland, https://dcenr.maps.arcgis.com/apps/MapSeries/index.html?appid=6304e122b733498b99642707ff72f754 or Australia, [18]) on grids with 250 m node spacing or less, Norway still lacks a complete coverage of the country. Almost 50 % of the country have been covered by high resolution airborne gamma radiometric surveys, partly over regions with expected high mineral potential but low population density. Additionally, thousands of ground-borne gamma radiation measurements are available, acquired primarily along roads as laboratory measurements on collected samples. As an important step towards a nationwide radiation risk assessment, we compute Uranium concentration maps of Norway by fusing the information [19] content of airborne and ground-borne Uranium measurements, respectively, with other densely mapped data covering Norway. The information fusion problem is set up as a multiple non-linear regression problem [20,21]. Since most of the data sets are delivered without data uncertainty, a propagation of the data uncertainty through the regression [22] is not possible. Model-related uncertainty, e.g., with regard to the optimal definition of regression model parameters, is taken into account by employing a Monte Carlo strategy [23]. Numerous algorithms for non-parametric multiple non-linear regression exist in parallel, which are all suited for the purpose intended here. However, these algorithms may perceive the data and their unknown uncertainty differently, they may respond differently to non-optimal model setting definitions, and may thus be regarded as different digital experts all qualified to tackle the given problem, but with an individual component in its solution. To overcome algorithm-specific aspects, we employ multiple digital algorithms in parallel mimicking an expert elicitation [24] to account for ontological uncertainty [25] related to the choice of our regression agent. Utilizing the suggested concept of a digital expert elicitation, we compute for the first time probabilistic ensembles of uranium concentration maps of Norway with quantified model and method related uncertainty discretely illustrated by the computed scenarios in our Monte Carlo and expert elicitation approach. In addition to the regression-based analyses, we employ a classification-based approach (e.g., [26]) to judge the risk for the local occurrence of spatially small but high uranium concentrations by integrating the ground-borne uranium measurements with the dense data sets used in the regression analysis, too.

We visually integrate the different computed uranium map scenarios into a loss-free static visualization concept suggesting digitated glyphs (e.g., [27]) to ease the perception of the map computation uncertainty when contemplating the maps on different spatial resolutions.

We begin by introducing the database considered. This is followed by describing the used non-parametric multiple non-linear regression algorithms and how they are embedded into a Monte Carlo approach and a digital expert elicitation. After discussing the employed classification approach, we show and discuss the results of our probabilistic uranium mapping approach covering Norway.

## 2 Database

### 2.1 Airborne uranium concentration data

The 10.13039/501100008647Geological Survey of Norway (10.13039/501100008647NGU) over the years 1976–2021 has collected Airborne Gamma-ray Spectrometry (AGRS) data through various government and commercial funded projects that cover approximately half of the country. AGRS data are collected by a mix of helicopter-borne and fixed wing surveys. Each AGRS sampling point is a weighted average value of total radiation originated from an oval shaped area – about 160 m in width and 180 m in length – of the ground surface with flying height, speed, and sampling rate of ca. 80 m, ca. 80 km/h, and 1 Hz, respectively. The AGRS data processing follows the general guidelines of International Atomic Energy Agency (IAEA) [28,29] and is well documented in various individual survey reports available at the NGU (www.ngu.no). The final outcome of the radiometry processing from each survey area is concentration of potassium, thorium and uranium in rocks and soils within 30–50 cm of the subsurface. Radiometric data from old surveys were reprocessed if data quality was not good and then stitched together to produce a complete compilation of uranium (U) in a single grid.

**Fig. 1:**
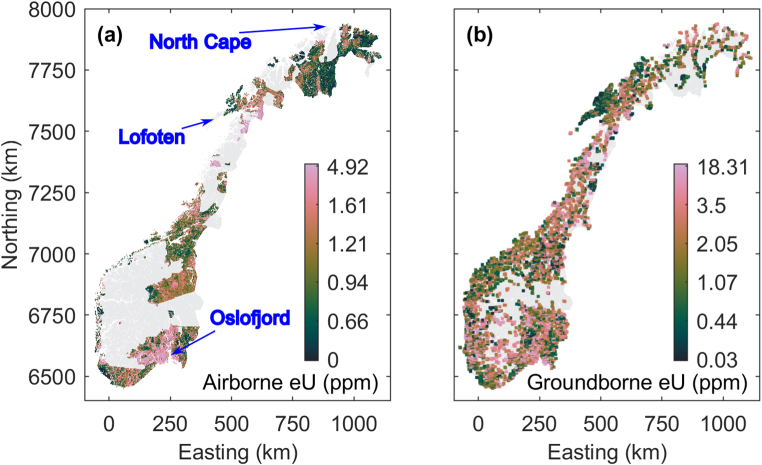
Available (a) airborne and (b) ground-borne Uranium measurements in Norway. Airborne data have been mapped on a regular grid with 250 m node spacing. Ground-borne data are depicted by squares with approximately 10 km side length for better visual perception. In (a), blue arrows mark prominent geographical locations for better orientation labeled in blue font.

Some of the old radiometric data existed in counts per seconds (cps) of U spectral window. However, newer radiometric data are presented in ground concentration of U in parts per million (ppm). The radiometric U grids from different surveys are leveled using a regression analysis between overlapping areas. A base and slope coefficient are calculated assuming newer U grids (in ppm) as a reference to recalculate older U grids (in cps) in ppm. Details about the regression analysis and the strategy can be found in Refs. [[30], [31], [32]]. In addition, few surveys were levelled using an improved version of the histogram matching algorithm by Ref. [33]. In case of histogram matching, overlapping areas were used in the histogram matching scheme to define a relation between the histograms of the two grids considering the newest survey as a reference and modifying the other grid [34]. There are some old survey areas without an overlap with new survey areas. In such cases, regression parameters from another survey area performed in the same year are used to level such areas. Subsequently, levelled grids are merged using the GridKnit module by Geosoft [35]. This process was repeated until the complete compilation is achieved to generate a single grid for a whole region. The spatial resolution of this grid is 50 m. We down-sampled it to 250 m grid node spacing to use it with other data sets provided on national scale. We expected a global uncertainty in the sense of a standard deviation of approximately 10 %. The compiled map is shown in Fig. 1a and referenced to EPSG 25833, like all maps shown in the following.

### 2.2 Ground-borne uranium concentration data

In total 11 110 ground-borne U data have been compiled from records available at the NGU. Measurements have been collected with rather irregular sampling intervals ranging from a few hundred meters up to more than 10 km, shown in Fig. 1b. The samples were acquired through drilling a 3-m-long core to ensure fresh, un-weathered bedrock, and their chemical compositions, including the uranium concentration, were analyzed in the NGU lab using inductively coupled plasma mass spectrometry (ICP-MS) technology. The minimum detectable concentration of the measurements varies between 0.3 and 0.01 ppm, and is dependent on the detection limit of the ICP-MS instrument which has been upgraded a few times over the years.

### 2.3 Densely mapped data covering Norway

**Fig. 2:**
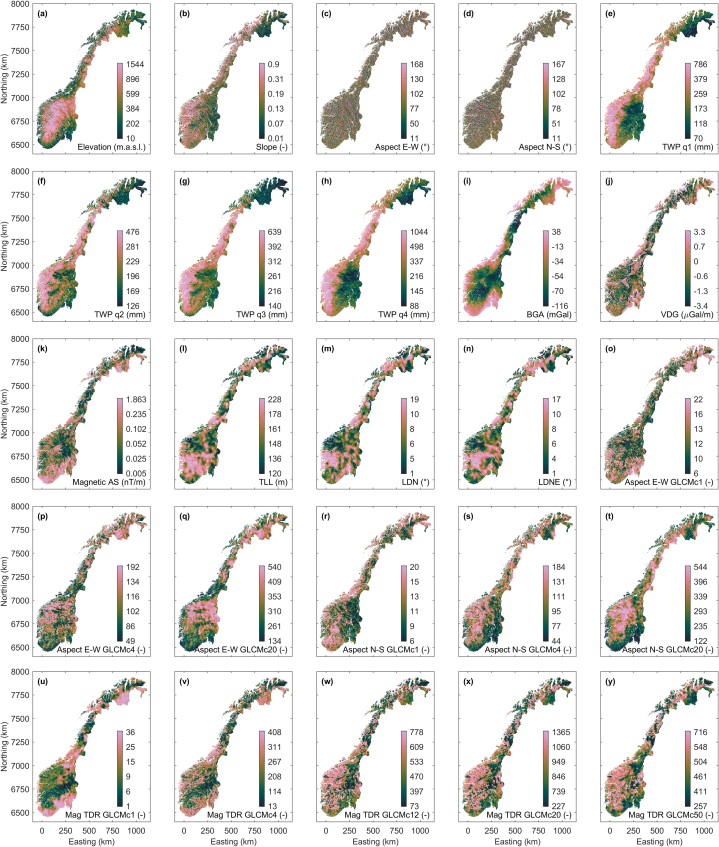
Maps of various state variables covering Norway on a regular grid with 250 m node spacing. All mapped state variables come as continuous (real-valued) variables. See Table 1 for further explanation.

**Fig. 3:**
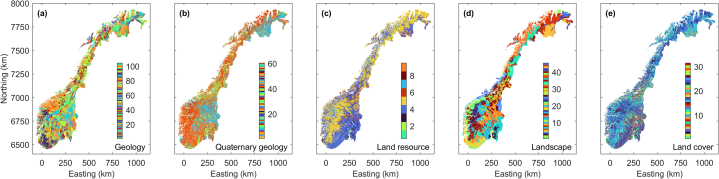
Maps of various state variables covering Norway on a regular grid with 250 m node spacing. All mapped state variables come as categorical variables. See Table 1 for further explanation.

In addition to the sparse airborne and ground-borne uranium data shown in Fig. 1, we consider further data sets mapped on a regular grid with 250 m node spacing. These maps cover entire Norway and provide dense information also in areas where larger gaps exist in the uranium data sets. In Fig. 2, Fig. 3, maps of various environmental state variables are shown that are provided as continuous (real-valued) and categorical (label) variables, respectively. Table 1 provides further information about data sources, data access, and the used type of the data for each map shown in Fig. 2, Fig. 3.

**Table 1:** Information about the maps shown in Fig. 2, Fig. 3. All maps shown in Fig. 2, Fig. 3 (feature sets in Table 2) are available on request from the authors.

Figure	Feature	Derived from data/feature set	Variable type
2a	Elevation	DTM 50, Norwegian Mapping Authority	continuous
2b	Slope, Topographic slope	Elevation (Fig. 2a)	continuous
2c	Aspect E-W, East-West component of Topographic Aspect	Slope (Fig. 2b)	continuous
2d	Aspect N–S, North-South component of Topographic Aspect	Slope (Fig. 2b)	continuous
2e	TWP q1, Total water precipitation 2018, 1st quarter	Monthly total water precipitation, Norwegian Meteorological Institute	continuous
2f	TWP q2, Total water precipitation 2018, 2nd quarter	Monthly total water precipitation, Norwegian Meteorological Institute	continuous
2g	TWP q3, Total water precipitation 2018, 3rd quarter	Monthly total water precipitation, Norwegian Meteorological Institute	continuous
2h	TWP q4, Total water precipitation 2018, 4th quarter	Monthly total water precipitation, Norwegian Meteorological Institute	continuous
2i	BGA, Bouguer gravity anomaly	Grav500, Norwegian Geological Survey	continuous
2j	VDG, 1st vertical derivative of gravity	1VDgrav500, Norwegian Geological Survey	continuous
2k	Magnetic AS, Magnetic analytical signal	Analytical Signal, Norwegian Geological Survey	continuous
2l	TLL, Total lineament length	Lineament map, Norwegian Geological Survey	continuous
2 m	LDN, Lineament deviation from North	Lineament map, Norwegian Geological Survey	continuous
2n	LDNE, Lineament Deviation from North-East	Lineament map, Norwegian Geological Survey	continuous
2o	Aspect E-W GLCMc1	Aspect E-W (Fig. 2c)	continuous
2p	Aspect E-W GLCMc4	Aspect E-W (Fig. 2c)	continuous
2q	Aspect E-W GLCMc20	Aspect E-W (Fig. 2c)	continuous
2r	Aspect N–S GLCMc1	Aspect N–S (Fig. 2d)	continuous
2s	Aspect N–S GLCMc4	Aspect N–S (Fig. 2d)	continuous
2t	Aspect N–S GLCMc20	Aspect N–S (Fig. 2d)	continuous
2u	MAG TDR GLCMc1	Magnetic tilt derivative, Norwegian Geological Survey	continuous
2v	MAG TDR GLCMc4	Magnetic tilt derivative, Norwegian Geological Survey	continuous
2w	MAG TDR GLCMc12	Magnetic tilt derivative, Norwegian Geological Survey	continuous
2x	MAG TDR GLCMc20	Magnetic tilt derivative, Norwegian Geological Survey	continuous
2y	MAG TDR GLCMc50	Magnetic tilt derivative, Norwegian Geological Survey	continuous
3a	Geology	Berggrunn 1:250 000, Norwegian Geological Survey	categorical
3b	Quaternary Geology	Løsmasse, Norwegian Geological Survey	categorical
3c	Land resource	Arealressurskart 1:250 000, Norwegian Institute of Bioeconomy Research	categorical
3d	Landscape	Landskapsregion, Norwegian Institute of Bioeconomy Research	categorical
3e	Landuse	CORINE Landcover 2012, Norwegian Institute of Bioeconomy Research	categorical

Fig. 2a–d shows topographic feature maps. The elevation map (Fig. 2a) has been derived from a digital terrain model with 50 m spatial resolution. Topographic slope (Fig. 2b) and aspect have been derived from the elevation map. The circular nature of topographic aspect, i.e., that minimal and maximal aspect of 0° and 360° are maximally similar, makes this data set different from slope and elevation data described on a linear axis where minimal and maximal values in the map indicate maximally dissimilar topographic states. To convert circular data onto linear axes, we split the topographic aspect data into their cosine and sine components highlighting east-west and north-south components of the slope direction (Fig. 2c and d).

In addition to topographic feature maps, Fig. 2e–h shows total water precipitation maps for the quarters of the year 2018, respectively. We use these maps as information layers about the rough trends in the annual near-surface water distribution. However, annual variations may exist within the time period of uranium data acquisition. Fig. 2i and j shows maps of Bouguer gravity anomaly and the first vertical derivative of gravity data. Both maps have been produced by upsampling maps projected on a grid with 500 m node spacing and applying linear interpolation. Fig. 2k shows the magnetic analytical signal map. The maps in Fig. 2l-n are derived from information about mapped lineaments. Lineaments are discrete features well distinguishable from each other and we interpolated the length of the mapped lineaments on a 250 m grid (Fig. 2l) as well as the local lineament deviation from north and north-east (Fig. 2m and n).

Fig. 2o-y shows the results of applying a gray-level co-occurrence matrix (GLCM) filter (e.g., [36]) to topographic aspect (Fig. 2o-t) and magnetic tilt derivative data (Fig. 2u-y). By applying the GLCM filter we highlight image texture information present in the original maps. Settings chosen for GLCM computation are given in Table 2. Using a sliding window technique, we compute GLCMs over squared subsections of the data grid. To achieve a rotationally invariant filter result we rotate the working direction of the filter stepwise and repeatedly compute GLCMs for different offsets. We compute the scalar intensity contrast (also known as inertia; e.g., [36]) for each GLCM and sum them over all GLCMs resultant from the same data subsection, i.e., sliding window. The filter result, the summed contrast, is assigned to the center pixel of the considered data subsection. We produce different GLCM filtered maps from the same input data set by applying the GLCM filter with different empirically chosen offset lengths. A small offset length highlights high-frequency image texture information. The maps in Fig. 2o-y shows the rotationally invariant intensity contrast of the computed GLCM matrices for different offset lengths.

**Table 2:** Settings employed for gray-level co-occurrence matrices (GLCM) computations applied to topographic aspect data (Fig. 2c and d) and magnetic tilt derivative data. Fig. 2o-y shows GLCM filter results depicting the contrast of the stacked GLCM matrices.

Figure	Feature	Window size (grid nodes)	Gray levels	Offsets (dx,dy) in grid nodes
2o, 2r, 2u	Aspect E-W GLCMc1, Aspect N–S GLCMc1, MAG TDR GLCMc1	11 x 11	10	(0,1), (1,1), (1,0), (−1,1)
2p, 2s, 2v	Aspect E-W GLCMc4, Aspect N–S GLCMc4, MAG TDR GLCMc4	21 x 21	10	(0,4), (1,4), …, (4,4), (4,3), …, (4,-4), (3,-4), …, (1,-4)
2q, 2t, 2x	Aspect E-W GLCMc20, Aspect N–S GLCMc20, MAG TDR GLCMc20	41 x 41	10	(0,20), (2,20), …, (20,20), (20,18), …, (20,-20), (18,-20), …, (2,-20)
2w	MAG TDR GLCMc12	21 x 21	10	(0,12), (2,12), …, (12,12), (12,10), …, (12,-12), (10,-12), …, (2,-12)
2y	MAG TDR GLCMc50	61 x 61	10	(0,50), (2,50), …, (50,50), (50,48), …, (50,-50), (48,-50), …, (2,-50)

The maps in Fig. 3a–e are presented as categorical variables and outline piecewise homogeneous regions describing patterns of mapped geology, quaternary geology, land resources, landscape, and land use, respectively. In the visualization shown here, we replace the original class labels, which assign geoscientific meaning to the mapped pattern, by numbers. The lowest number is always 1, and the highest number equals the number of different classes defined in the map. While a geoscientist may consider this representation as incomplete, it is sufficient to represent the information used in our information fusion approach, which does not consider the geoscientific meaning of different class labels. Readers interested in the geoscientific labels of these maps are referred to the openly available online publications of these maps offered by the providers named in Table 1. Analogue to the precipitation data in Fig. 2, the land use data considered represent only a single year from the uranium data acquisition period. Other than the maps in Fig. 2 mapped on a linear axis, there exists no ordering of the different numeric class labels along the axis, i.e., the numeric difference of two class labels is not related to the similarity (or distance) of the described natural states.

A soil map would have been a useful addition to the database but there is a lack of national soil property maps in Norway [37]. Norway has some soil cover but exposed bedrock at most places. When soil is derived from bedrock underneath then it is likely to have similar radiation as the exposed bedrock. If the soil cover is locally thick and transported by external agents, e.g., wind, deglaciation etc., then it might have different radiation characteristics than the underneath bedrock. In most cases the soil will be fully or largely water saturated and might be correlated with low radiation areas. To understand whether detected low radiation is due to absence of uranium rich bedrock or thick soil cover is not the objective of this paper. Note that we used a quaternary geology map which has thin and thick soil cover aspects and it doesn't show any significant importance in the feature analysis.

## 3 Methodology

### 3.1 Uranium map computation by regression

#### 3.1.1 Information fusion by multiple non-linear regression

We employ multiple non-linear regression (e.g., [[20], [21]]) to reduce the epistemic uncertainty [38] in the uranium data sets, i.e., uncertainty associated with (knowledge) gaps in the maps shown in Fig. 1. A multiple regression model is a function *g* linking a one-dimensional response variable ***Y*** with a multi-dimensional predictor variable ***X*** and a multi-dimensional disturbance variable ***U***, ***Y*** = *g*(***X***, ***U***) (e.g., [39]). Here, airborne or ground-borne uranium concentration (Fig. 1) is the response variable and the predictor variable comprises the data sets imaged in Fig. 2, Fig. 3. Since we lack quantitative information about the uncertainties associated with the data sets in ***X*** and ***Y***, we consider ***U*** = 0. This results in the ignorance of data related uncertainty and uncertainty propagation when finding a regression model and makes learning *g* from known co-located instances of the data in ***X*** and ***Y*** a deterministic task for a given deterministic learning methodology.

We employ regression algorithms using least-squares fitting (e.g., [40]) of data and model response to optimize their objective function in a metric space. Hence, we have to preprocess labeled maps composed of categorical variables (Fig. 3). We transform the categorical data sets into multi-dimensional binary dummy variables using one-hot encoding (e.g., [41]). We scale the continuous variable data sets (Fig. 2) and the dummy variables according to Gower's coefficient of generalized dissimilarity [42] to achieve a multi-dimensional predictor variable suitable for regression algorithms employing Euclidean distance measures in their objective function.

#### 3.1.2 Uncertainty quantification by digital expert elicitation

Multiple non-linear regression can be seen as a supervised task of learning *g*, or parameters (coefficients) of a predefined *g*, from co-located samples of ***X*** and ***Y*** [43]. Because of lacking knowledge about the expected shape of *g*, we decide for a non-parametric solution of the regression problem. Various algorithms are known to be able to act as universal function approximators including Multi-Layer Perceptrons (MLP; e.g., [44]) as a distinct architecture of feed-forward artificial neural networks with one or more hidden layers, random forests (RF; [45]) and support-vector machines (SVM; [46]).

In principle, all these algorithms can solve a regression problem correctly and their utilization is generally equally justified, but differences exist. For example, for a regression tree employed in a random forest the considered working hypothesis relies on splitting the considered predictor variable into a number of discrete, non-overlapping groups, represented by the tree's leaf nodes. In contrast, MLPs can mimic regression trees when using discrete activation functions (e.g., [47]), but it is more common to use MLPs with continuous activation functions leading away from the splitting concept and the utilization of discrete non-overlapping groups. Working with discrete data of limited precision, splitting-based and continuity-based working hypotheses are equally acceptable for our study.

Practical differences between universal function approximators may exist depending on the parametrization of the algorithm and the data perception, e.g., with regard to the handling of inter- or extrapolation parts in the regression problem, uncertainty propagation, or impacts of data overfitting on the learned regression model. This may have stimulated some authors to do empirical comparisons of different regression algorithms on a given database (e.g., [48]). Such comparisons could not claim generality beyond the studied database and are not of benefit to us here. Which algorithm is the most suited one, and decisions about optimal algorithmic parameter settings for a task, require a deep mathematical analysis of the faced regression problem including knowledge about data uncertainty, which is not available for the considered database.

In our situation, we consider universal function approximator realizations, e.g., as available in R, Python, or Matlab toolboxes, as equally qualified solutions to learn a regression model from our data. Mimicking an expert elicitation (e.g., [24]), we consider available realizations of different algorithms for non-parametric multiple non-linear regression analysis as (digital) agents equally qualified to do the same task, even if their regression results may slightly differ due to “individual” (algorithm-specific) peculiarities. We therefore employ MLP and RF in parallel as qualified agents in a digital expert elicitation of our regression problem. The RF follows a splitting-based working hypothesis, and we employ the MLP network following a continuity-based working hypothesis using fully connected layers with Rectified Linear Unit (ReLU; e.g., [49]) activation functions.

When training the RF and MLP algorithms, the fraction of the input data considered for sampling is 0.63 of the total number of training data. Since optimal settings for the RF and MLP are not precisely known, we vary some algorithmic settings empirically and select by posterior analysis what appears appropriate. We begin with the RF, and narrow the tested parameter variability for the MLP taking the data misfit range of accepted RF regression models as rough guideline when setting up the MLPs, so that the MLP regression model misfit range approximately matches those of the RF regression models. To account for uncertainty arising from randomized selections within the training of RF and MLP algorithms, we repeatedly train them 10 times using different initialization states of the random number generator. Thus, we implement a Monte Carlo approach quantifying regression model uncertainty for RF and MLP models into our digital expert elicitation.

Our approach results in uranium concentration maps covering Norway on a regular grid with 250 m node spacing. We achieve an ensemble of acceptable maps, illustrating possible scenarios. These ensembles are discrete realizations of a probability density distribution accounting for uncertainty with regard to the selection of “the right” or optimal regression methodology and some aspects of regression model uncertainty due to internal random choices of each algorithm. Data uncertainty and its propagation into the final uranium concentration maps are ignored and might result in an overly optimistic uncertainty assessment. However, a Monte-Carlo based propagation of data uncertainty through multiple non-linear regression (e.g., [22]) could be straight forward combined with the digital expert elicitation used here to realize a more all-embracing uncertainty analyses, if uncertainties for response and predictor variables would be available. Additionally, practical impossibility to address all decision points in a methodology may also result in overly optimistic uncertainty estimation. For example, even within a given MLP network architecture, the utilization of different activation functions might be possible, which has not been considered in this study, but might have resulted in potentially more extreme uranium concentration estimations at some map locations than those found by our endeavors.

### 3.2 Uranium map computation by classification

We employ a RF algorithm to classify the Norwegian territory into four classes outlining regions with different occurrence of locally high uranium concentrations. The number of classes is empirically chosen. Therefore, we integrate the ground-borne uranium measurements (Fig. 1b) with the dense data sets (Fig. 2, Fig. 3). In analogy to the regression analysis, we preprocess and scale the maps in Fig. 2, Fig. 3 and use them as predictor variable. If multiple uranium measurements are located within the same grid cell of the densely mapped data sets, we only consider the reading with the highest uranium concentration. The total training data set for learning a classification model comprises 8820 ground-borne uranium concentration measurements associated with the predictor variable. We split the ground-borne uranium concentration data into four groups with the ranges [0, 10), [10, 20), [20, 50), and [50, ∝) ppm referred to as classes 1, 2, 3, and 4, respectively. They comprise 8446, 240, 113, and 21 samples, respectively. Note that more than 99.5 % of the available training samples fall into class 1. We repeatedly train RF classification models employing 1000 decision trees with a minimal leaf node size equal to one. When learning the classification model, the fraction of the input data considered for sampling with replacement is 0.63 of the total number of training data. To compensate for the high number of samples in class 1, we employ different class prior probabilities for random sample selection underemphasizing the importance of class 1. Additionally, we also vary the definition of the cost matrix of the algorithm from equal penalization of miss-classification towards higher penalties for assigning samples to a class lower than they would naturally fall in.

## 4 Results and discussion

### 4.1 Random forest regression

#### 4.1.1 Analysis of training performance

**Fig. 4:**
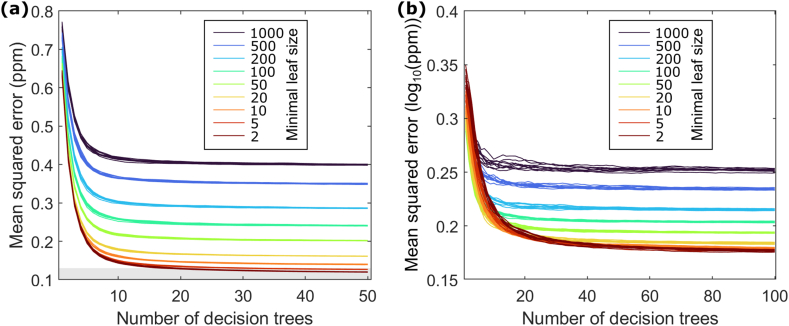
Random forest performance when trained on (a) airborne and (b) ground-borne Uranium measurements for different number of decision trees in the forest and depths of the decision trees. For each Random Forest architecture, 10 models have been trained with different states of the random number generator resulting in 10 performance lines. The gray box in (a) marks the empirically estimated mean uncertainty of the airborne Uranium map.

We employ a random forest regression algorithm taking the airborne uranium data as response variable. We evaluate the training performance of the RF by computing the out-of-bag prediction error measured by the mean-squared error over the number of decision trees in the RF (Fig. 4a). For all computed regression models, stable convergence was achieved for RFs comprising at least approximately 30 decision trees. Increasing the depths of the decision trees considered in the RF by lowering their minimal leaf node sizes leads to regression models associated with smaller MSE (Fig. 4a). The estimated global mean relative data uncertainty of 10 % for the airborne uranium data corresponds to an MSE of approximately 0.127 ppm. The gray box in Fig. 4a depicts this threshold. RF regression models with minimal tree leaf sizes of 5 or lower reach model fits slightly better than the assumed airborne uranium data uncertainty (Fig. 4a). This gives us confidence that the general flexibility of the RF model and the employed predictor variable information are suitable to explain the airborne uranium data sufficiently well. Refinement of the RF regression models beyond minimal leaf node sizes of 20 leads only to small reductions of the models’ MSE errors. This makes us believe, that the major trends in the relation between predictor and response variable can be explained by RF regression models comprising decision trees with a minimal leaf node size of 20. Further model refinement may increasingly result in data overfitting. Since data uncertainty is not quantitatively known, we cannot define a data-related stopping criterion when training the RF regression model. If the number of training data is limited and a low number of decision trees is used, the found RF regression model may depend on random selections within the bootstrapping and feature selection procedures followed during the RF training. We evaluate this dependency by repeatedly training 10 RF regression models comprising decision trees with identical minimal leaf node sizes but different initializations of the random number generator before starting the RF training procedure. This results in 10 model performance curves for each leaf node size (Fig. 4a). These curves are not fully coincident but highly similar, indicating a high robustness with regard to randomized algorithmic decisions during the training procedure.

Analogue to the analyses shown in Fig. 4a, we do the same using the ground-borne uranium data (Fig. 1b) as response variable. The results are shown in Fig. 4b. Due to the strongly skewed distribution of the ground-borne uranium data, with only a few very high uranium concentration measurements, we train the RF models after applying Brigg's logarithm to the ground-borne uranium data. We find RFs comprising at least 70 decision trees to be sufficient to achieve stable convergence. Utilization of decision trees beyond a minimal leaf node size of 50 might increasingly suffer the risk of data overfitting. Due to the smaller training data set, the dependency on random selections with regard to bootstrapping and feature selection are stronger than observed for the airborne uranium training data set, but still sufficiently stable to produce grouped training performance curves.

#### 4.1.2 Analysis of feature importance

**Fig. 5:**
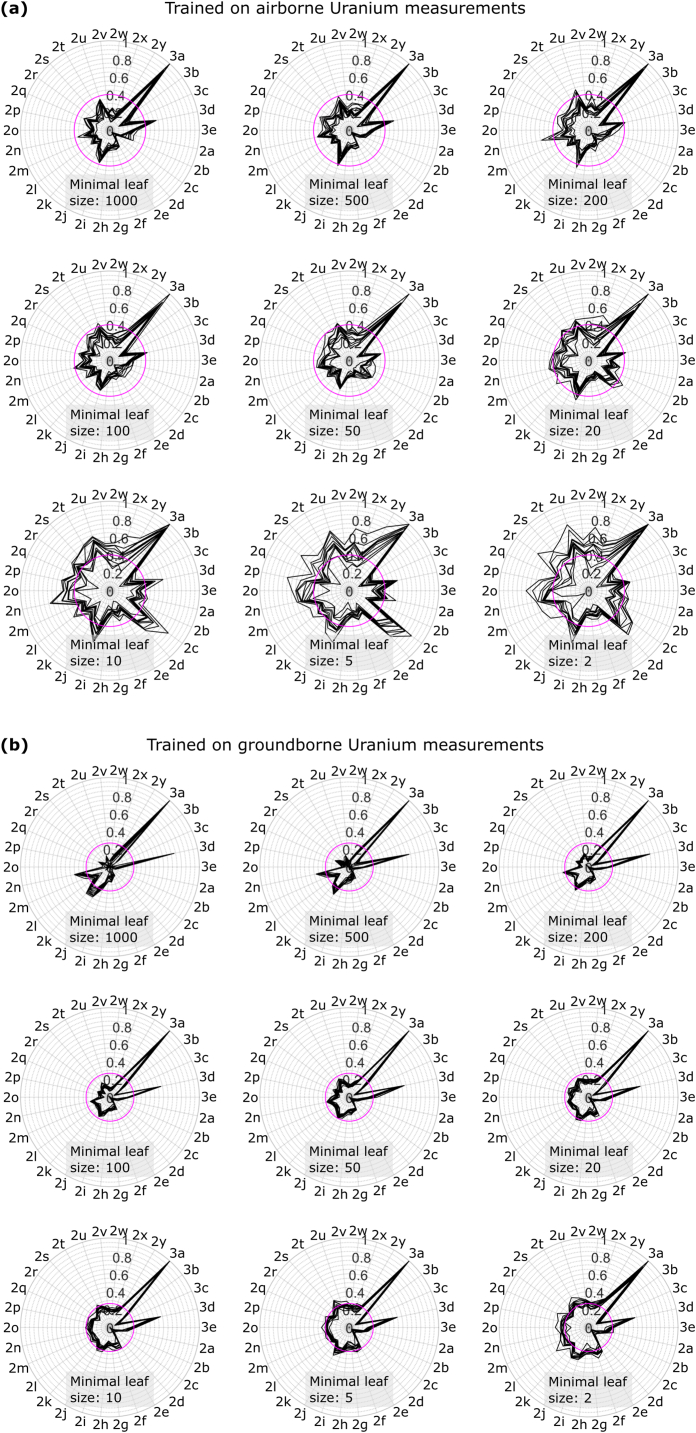
Normalized variable importance measure (VIM) when learning a Random Forest prediction model using (a) airborne and (b) ground-borne Uranium data. VIM has been repeatedly done for 10 RF trainings with different states of the random number generator per forest illustrate by 10 black polygons per rose diagram. Magenta circles for visual guidance, see text for further explanation. Azimuthal labels refer to the state variables in Fig. 2, Fig. 3 by their subplot labels.

Fig. 5a shows the out-of-bag feature importance estimates by permutation [45] associated to the random forest models with different leaf node sizes trained using the airborne uranium data. We display the feature importance using polar plots. Each polar plot shows the results for 10 RF models with different random number generator initialization for a fixed minimal leaf node size. The angle information is related to the data sets shown in Fig. 2, Fig. 3 indicated by the Figure numbers (see also Table 1). Feature importance grows linearly with the distance from the center of the polar plot. We normalize the feature importance for each polar plot to the maximal feature importance value. The geological map (Fig. 3a) is found to be the most important feature regardless of the refinement level, i.e., the minimal leaf node size, of the regression model. However, its relative importance is maximal for coarse regression models with minimal leaf node size of 1000 and reduces with decreasing leaf size (Fig. 5a), i.e., ongoing model refinement by growing the decision trees in the RF further. This means that the early definition of the regression model is largely controlled by the mapped geology. Since uranium data are not available for all mapped geological classes, the application of these geology-dominated regression models will result in high fractions of extrapolation over some parts of Norway with mapped geology not considered in the training data. Other features of increased prediction importance when considering rather coarse models are related to landscape information (Fig. 3d), geophysical gravity and magnetic data (Fig. 2i and u, respectively), and the occurrence of mapped lineaments (Fig. 2n). A reverse trend is observed for the topographic aspect (Fig. 2c and d). Topographic aspect importance grows slowly when employing decision trees with leaf node sizes between 1000 and 50. For leaf node sizes below 50, its importance starts to grow quickly reaching a plateau for leaf node sizes of 5 and 2. These data are predominantly used in the later decisions when training the RF models and almost completely ignored in the early training phase. Topographic aspect maps exhibit the lowest correlation lengths among the data shown in Fig. 2. This makes these data particularly suitable for fine adjustments at distinct locations in the maps, but a general relation between topographic aspect and uranium concentration seems to be absent, because of the ignorance of these data in the early splitting decisions in the trees of the RF models. Here, the short-wavelength spatial information provided by topographic aspect takes the role of a stochastic field offering arbitrary options for the regression model to accommodate very local effects in the uranium data. This trend starts to dominate for models comprising decision trees with leaf node sizes of 20 or lower strengthening the indication that regression models with leaf node sizes smaller than 20 might suffer overfitting. This indication is also supported by a growing dependency of the feature importance on random bootstrapping and aggregation effects when training the RF models. For RF models with minimal leaf node size of 20 or smaller, the feature importance shows strong dependence on random effects in the training of the RF models, and only mapped geology (Fig. 3a) and lineaments (Fig. 2n) remain clearly above a relative feature importance of 0.4 (magenta circle in Fig. 5a).

Analogue we analyze the feature importance when using ground-borne uranium data as response variable (Fig. 5b). Again, features of increased prediction importance when considering rather coarse models are related to mapped geology (Fig. 3a), landscape information (Fig. 3d), geophysical gravity and magnetic data (Fig. 2j and k, respectively), and the occurrence of mapped lineaments (Fig. 2n). This confirms a reasonably similar physical relationship between the information contained in the predictor variable and the response variable independent of the spatial integration area of the uranium data. A ground-borne uranium datum comprises information integrated over an area up to a few square meters, whereas an airborne uranium datum represents a map area of 62500 m^2^. As observed for the airborne data, RF model refinement results in an increasing loss of discriminative power of the feature importance analyses resulting in mapped geology (Fig. 3a) and landscape information (Fig. 3d) as the only remaining features of significantly higher importance than 0.26 (magenta circle in Fig. 5b) when growing the decision trees in the RF model to minimal leaf node sizes of 2. Opposite to the airborne uranium data, topographic aspect (Fig. 2c and d) remains of low importance regardless of the model refinement (Fig. 5b). This may be due to the low spatial density of the ground-borne uranium sampling, where the average sampling distance is larger than the dominant correlation length in topographic aspect data. Therefore, the aspect data offer information about the spatial variability beyond the information content in the relatively sparse ground-borne data set.

#### 4.1.3 Uranium map estimation by applying the regression model

**Fig. 6:**
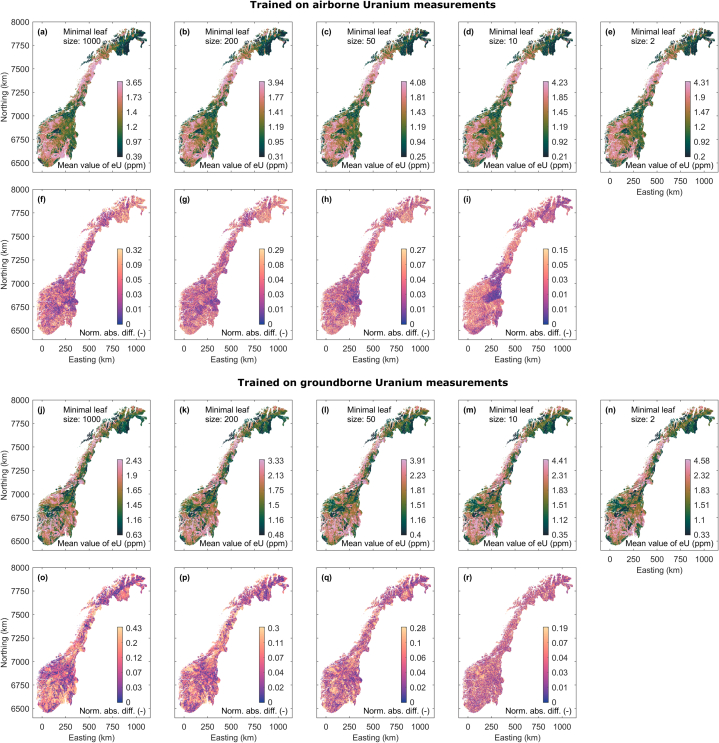
(a)–(e) Mean value Uranium maps achieved by averaging over 10 prediction models learned with different states of the random number generator for forests with different decision tree depths using the measured airborne Uranium data for training. (f)–(i) Normalized differences between the five maps in (a)–(e) in corresponding order. (j)–(r) analogue to (a)–(i), but for training on ground-borne Uranium.

We show uranium concentration maps in Fig. 6a–e for RF models trained using the airborne uranium measurements and different minimal leaf node sizes. The maps show mean values for each grid node computed from 10 uranium maps achieved by applying the 10 RF regression models learned with different random number generator initialization. The mean maps are highly similar and differences between them are hard to recognize for a human contemplator. Despite the poor data fit of regression models with leaf node size 1000 (see Fig. 4a), the mean predicted uranium concentration map shows already the same dominant pattern as all the maps achieved by models fitting the data better. Model refinement increases the total range of estimated uranium concentrations allowing for locally higher or lower prediction values without changing the long-wavelength information in the maps anymore. The pairwise differences between adjacent maps in Fig. 6a–e are shown in Fig. 6f–i. The maps in Fig. 6f–h shows a rather random distribution of differences between the maps up to a leaf node size of 10. The map in Fig. 6i shows the difference between maps associated to RF models comprising trees with minimal leaf node size of 10 and 2. Here, systematically lower differences between both maps exist in areas for which training data were available (cf. Fig. 1a). Such a systematic difference in prediction variability may indicate a systematic effect due to overfitting the given training data. Hence, we judge the boundary to overfitting exists somewhere between RF models with leaf node size 10 and 50.

In Fig. 6j-n we show uranium concentration maps achieved from RF models trained using ground-borne data as response variable. The corresponding pairwise difference maps are shown in Fig. 6o-r. The uranium concentration maps are again similar, but some differences are visible showing a stronger effect of model fine tuning on large-scale changes in the produced uranium maps. This may be an effect caused by the small amount of available training data. The difference reveals a rather long-wavelength pattern of regions with low and high differences when comparing maps produced from coarse RF models (Fig. 6o). For Northing coordinates between 6800 and 7100 km and above 7650 km, large regions of low differences exist. A clear relation of these areas to high or low training data density is not possible, but these patterns reduce to a more random behavior, so that we would judge that models with leaf node sizes larger than 200 do not allow for sufficient complexity, yet.

#### 4.1.4 Which maps to choose from the computed ensemble

A precise determination of a data uncertainty related optimal model fitness avoiding data overfitting is not possible, but our performance analysis based on model objective function, feature importance, and difference of the predicted maps gives some indications. When using the airborne uranium data as response variable, we consider the best RF model to be achieved for minimal leaf node sizes in the interval [20 100]. When using the ground-borne data set as response variable, we judge RF models with minimal leaf node sizes in the interval [50 200] as models offering stability with regard to feature importance and uranium map changes. These models compromise a good data fit while largely avoiding data overfitting.

### 4.2 Multi-layer perceptron regression

#### 4.2.1 Analysis of training performance

**Fig. 7:**
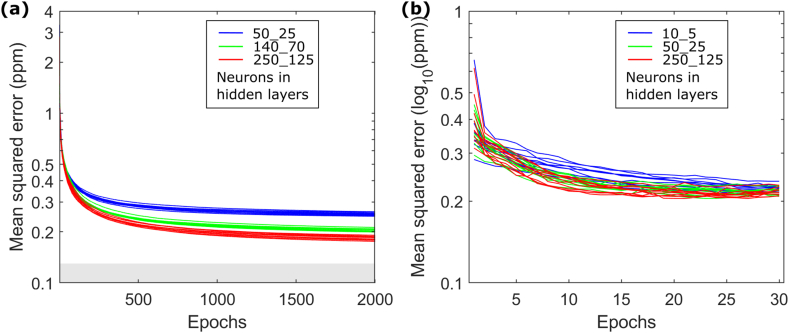
Multi-layer Perceptron performance when trained on (a) airborne and (b) ground-borne Uranium measurements. MLPs with two layers of hidden neurons have been employed and three different hidden layer sizes have been considered. For each MLP architecture, 10 models have been trained with different states of the random number generator resulting in 10 performance lines. The gray box in (a) marks the empirically estimated mean uncertainty of the airborne Uranium map.

We set up and trained MLPs using the airborne uranium data as response variable. Taking the RF model data fit for models with minimal leaf node sizes in the interval [20 100] as benchmark, we strived to employ MLPs offering comparable data fit. Based on empirical choice, we used MLPs comprising the data input layer, two hidden layers of neurons, and an output layer. The number of neurons in the hidden layers was empirically chosen with the second hidden layer comprising half the number of neurons of the first hidden layer. Fig. 7a shows the training performance of the MLPs for the airborne uranium data. Utilization of more neurons increases model complexity and allows for generally better data fit. After 2000 epochs of training, stable models are achieved. We repeatedly trained each MLP 10 times with different random number generator initialization. As for the RF models, a slight performance dependency on random components in the training procedure exists.

Analogue, we set up and trained different MLP regression models using the ground-borne uranium data as response variable. Due to the limited amount of training data, training performance varies stronger with random selections during the training phase (Fig. 7b). Model fitness of the complex models is only slightly better than those of the simplest model tested comprising only 10 and 5 neurons in its hidden layers.

#### 4.2.2 Analysis of feature importance

**Fig. 8:**
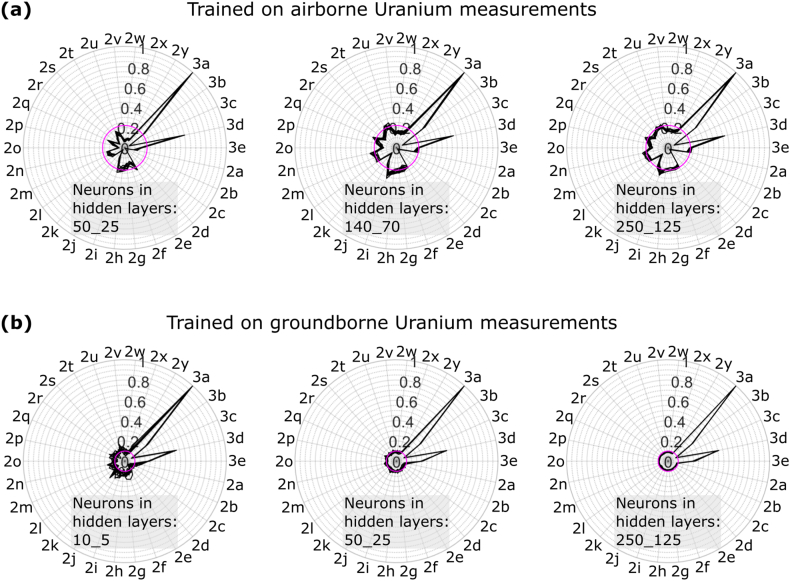
Normalized weights connecting the input layer with the first hidden layer in the MLPs for (a) airborne and (b) ground-borne Uranium training data. Normalized weights are shown for 10 MLP trainings with different states of the random number generator illustrated by 10 black polygons per rose diagram. Magenta circles for visual guidance, see text for further explanation. Azimuthal labels refer to the state variables in Fig. 2, Fig. 3 by their subplot labels.

We analyze the weights of the MLP between the input layer and the first hidden layer as approximation for the feature importance of the data sets in the predictor variable (e.g., [50]; Fig. 8). When taking the airborne uranium data as response variable, as for the RF models, geology (Fig. 3a) and landscape (Fig. 3d) information receive high weights (Fig. 8a). The maps shown in Fig. 2i and n score also high again, as known from the RF feature importance analysis, but differences exist, too. For example, the MLPs weight information based on precipitation data shown in Fig. 2e pretty high, which is of low importance in the training of the RF models. As known from the RF models, with increasing model refinement, i.e., growing number of neurons, many features receive increasingly equal weights.

Taking the ground-borne uranium data as response variable results in weights equal for most of the data sets in the predictor variable (Fig. 8b). Exceptions are again mapped geology and landscape information (Fig. 3a and d, respectively). This may point towards model overfitting by explaining small or local variations of the response variable randomly matching with local patterns in a data set in the predictor variable.

#### 4.2.3 Uranium map estimation by applying the regression model

**Fig. 9:**
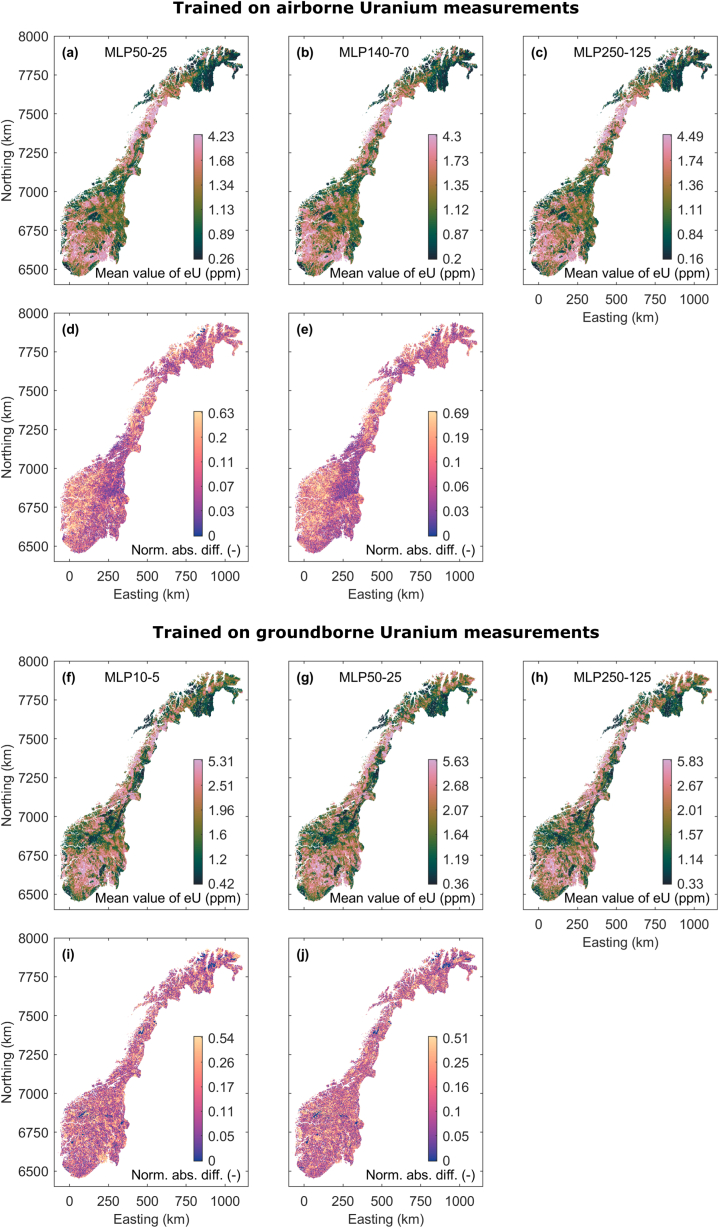
(a)–(c) Mean value Uranium maps achieved by averaging over 10 prediction models learned with different states of the random number generator for MLPs with different hidden layer sizes using the measured airborne Uranium data for training. (d) and (e) Normalized absolute differences between the maps in (a)–(c) in corresponding order. (f)–(j) analogue to (a)–(i), but for training on ground-borne Uranium.

We show the uranium map predictions achieved when applying the MLP regression models trained by airborne uranium data in Fig. 9a–c. For each map grid node, the mean uranium concentration over 10 MLP models achieved by random training initialization are shown. Again, the maps are highly similar but not identical. Pairwise difference maps between the uranium concentration maps are shown in Fig. 9d and e. The difference map in Fig. 9e starts to reveal a systematically lower difference in areas where training data are available. As for the RF maps, we take this systematic effect as a hint towards growing data overfitting. Fig. 9f–j shows the predicted uranium maps and their pairwise differences when training the MLPs with the ground-borne uranium data. The uranium maps are highly similar, and the difference show a random behavior with even lower correlation lengths when comparing the maps achieved by MLPs with higher model complexity.

#### 4.2.4 Which maps to choose from the computed ensemble

As for the RF results our MLP performance analysis is based on model objective function, feature importance, and difference of the predicted maps gives some indications. When using the airborne uranium data as response variable, we judge all three models as acceptable analogue to the matching RF models with minimal leaf node sizes in the interval [20 100]. When using the ground-borne data set as response variable, we face hints for overfitting for the more complex models with regard to model objective function and feature importance behavior. Therefore, we only consider the simple model we judge RF models with minimal leaf node sizes in the interval [50 200] as models offering stability with regard to feature importance and uranium map changes.

### 4.3 Random forest classification

**Fig. 10:**
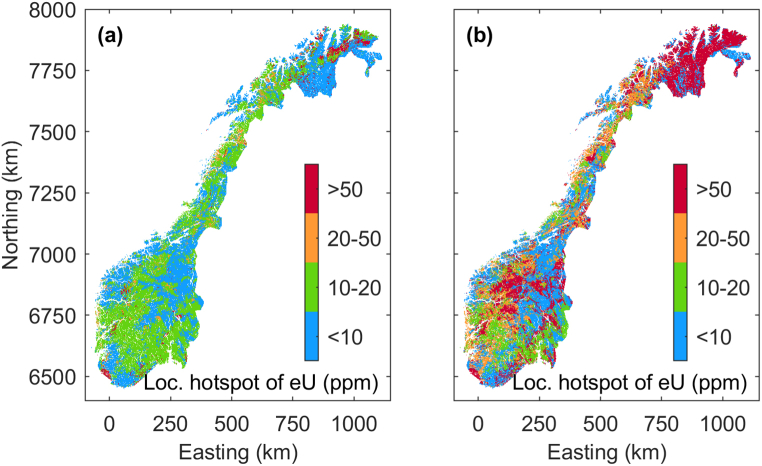
Risk map for occurrence of local hotspots resultant from a Random Forest classification of the ground-borne Uranium data. Note, due to the low number of training samples in the classes >20 ppm, different schemes have been used for oversampling values of high Uranium concentration in the training procedure and penalizing misclassification of high values during the training phase. (a) represents a conservative assessment, and (b) a rather alarmistic assessment. For more details see text.

We employed a random forest to classify the ground-borne uranium data, divided into four groups, using the same predictor variable as for the regression. We followed two different approaches to compensate for strongly different group sizes in the available training data. Fig. 10a shows a classified map of uranium concentrations achieved using a symmetric 4x4 cost matrix **C** = 1 – **I**, with **I** being the identify matrix. If the RF model assigns a datum in the validation data set to a group different than it belongs to, this causes costs of unity, regardless whether the datum had been assigned to a group associated with higher or lower uranium concentrations than the correct group. Additionally, we strongly oversample training data from the groups with low amount of data by setting the group prior probabilities in the training procedure to 0.05, 0.05, 0.1, and 0.8 for groups 1 to 4, respectively.

A more alarmistic result (Fig. 10b) is achieved when choosing group prior probabilities closer to the inverse of the number of samples in a group, e.g., 0.8, 0.1, 0.05, and 0.05. This still oversamples the groups 2–4 in the training process. Additionally, we now strongly penalize classification failures when assigning a datum to a group associated to lower Uranium concentrations than it would correctly fall in. The used 4x4 cost matrix **C** is column wise vectorized as vec(**C**) = [0, 101, 201, 301, 1, 0, 101, 201, 1, 1, 0, 101, 1, 1, 1, 0]. Both maps in Fig. 10 outline regions associated to the four groups reaching up to 10, 20, 50, or more than 50 ppm, respectively. Despite using the same data as in the regression, these maps show spatial distributions other than the uranium concentration maps achieved by regression. We consider them as a conservative and alarmist assessment of local uranium concentration hotspots, possibly even beyond the 250 m grid node spacing of the maps. It is a supplementary layer of information not striving to image the expected typical state but rather potential upper limits at local positions.

### 4.4 Visual integration of results

**Fig. 11:**
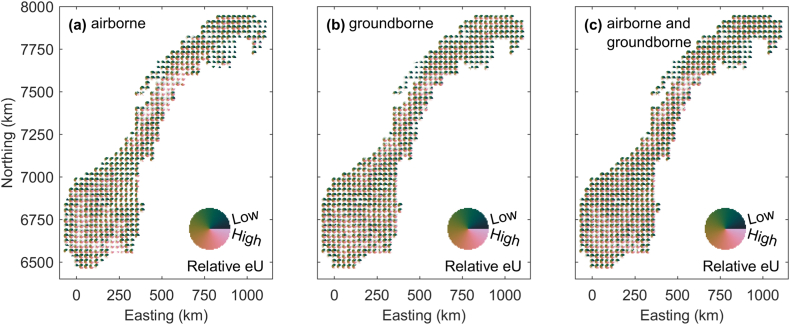
Overview probabilistic information about the predicted Uranium concentrations on a regular grid with 30 km node spacing for Random Forest and MLP results for (a) airborne and (b) ground-borne Uranium data considered for training the prediction models. (c) is the aggregation of (a) and (b). Since each prediction map was histogram equalized to overcome systematic differences between them, only a relative scale is provided.

**Fig. 12:**
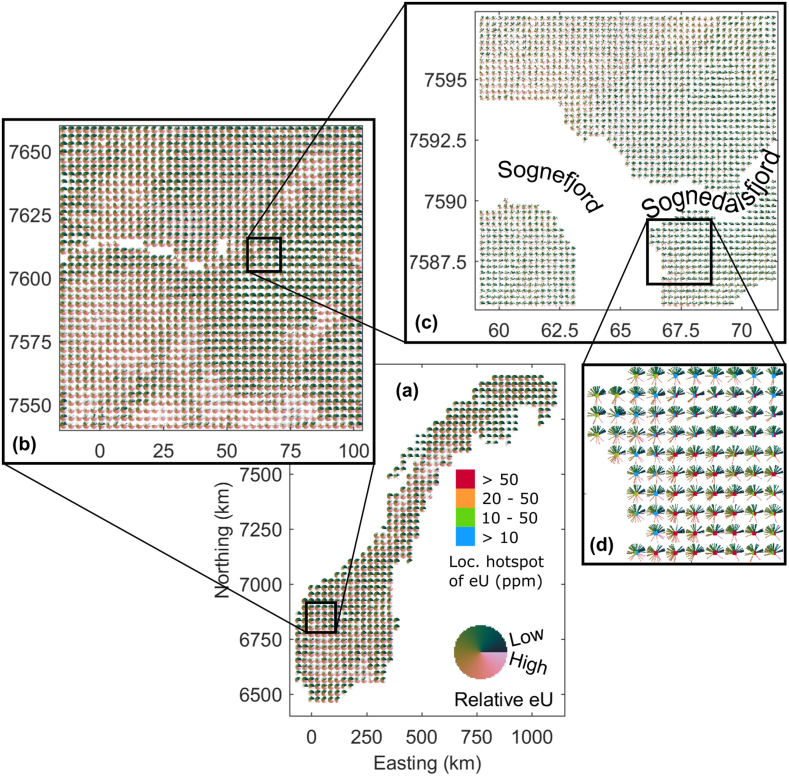
Zoom-in examples for regional and local scale assessment of the overview map shown in Fig. 11c, which is repeated here in (a). (b) Regional scale assessment example comprising approximately 14400 km^2^ on a regular grid with 3 km node spacing. (c) The local scale map example covers approximately 156 km^2^ on a regular grid with 250 m node spacing, which corresponds to the grid of the predictor variables in Fig. 2, Fig. 3 and defines the spatial resolution limit. (d) Enlarged image section of the map in (c). Now, information about the risk for local Uranium hotspots taken from the maps in Fig. 10a and b has been added by colored rectangular boxes in the center of each glyph. Left and right box correspond to Fig. 10a and b respectively.

We use digitated glyphs (see appendix) to integrate the probabilistic results of our uranium map computations (Fig. 11, Fig. 12). We visualize the results achieved by RF regression with minimal leaf node size of 50 and MLP regression with 140 and 70 neurons in the hidden layers (Fig. 11). Due to repeated training with different random number generator initialization, we have 10 uranium concentration maps from RF models and 10 from MLP models that are fully co-located. For each node on the 250 m grid underlying our computations, we achieved 20 possible uranium concentration states. We scale the 10 RF and the 10 MLP maps using histogram equalization. This allows only for a relative colormap common to all scenarios. However, for each computed map a quantitative colormap could be assigned to the glyphs. For each glyph we use a 40 x 40 grid cell mesh and coverage thresholds of 0.1. Fig. 11a shows the results achieved by RF and MLP regression models using airborne uranium as response variable. We chose a 30 × 30 km discretization thus integrating the information of 14400 map grid nodes into one glyph. On this coarse spatial resolution, many glyphs show a variability from low to high uranium concentration values. Nevertheless, for some regions a clear tendency towards the domination of higher or lower uranium concentrations can be observed. For example, east of the Lofoten (Easting of approximately 500 km, Northing of approximately 7500 km) uranium concentrations are generally high. Analogue to the airborne data, we produce a glyph map based on ground-borne uranium data for training RF models with minimal leaf node size of 100 and MLP models with 10 and 5 neurons (Fig. 11b). Again, many glyphs show a high variability and regions of predominantly high or low concentrations are partly different from those shown in Fig. 11a. The glyph concept allows to integrate the results shown in Fig. 11a and b to achieve a uranium concentration prediction map based on airborne and ground-borne training data (Fig. 11c). Glyphs become generally even less focused compared to the maps in Fig. 11a and b, which underlines the existence of differences between the prediction results achieved when taking airborne or ground-borne data as response variable.

Using the glyph-based visualization, limited display size and the need to image a vector of information per grid node may potentially require to lower the spatial resolution, i.e., to employ glyphs integrating several map grid nodes to enable a large-scale overview image. However, the chosen glyph technique generally allows for loss-free visualization of ensembles of co-located maps. Taking the overview map shown in Fig. 11c as a starting point, Fig. 12 shows different zoom levels down to the map grid scale (Fig. 12d). For Fig. 12a–b and c-d we use coverage thresholds of 0.1 and 0, respectively. In Fig. 12c and d, each glyph represents a map area of 250 × 250 m which equals the resolution limit and allows for detailed local analyses. Each glyph comprises 40 fingers. Furthermore, we display in the center of each glyph the information of the conservative and alarmistic classification results shown in Fig. 10.

Fig. 12 shows the uranium concentration uncertainty according to two different data-driven regression methodologies, i.e., digital experts, including aspects of regression model-related uncertainty for the RF and MLP based methodology. It could be straight froward extended to accommodate results of further regression methodologies. Further consideration of the results of other methodologies may increase the variability of the predictions, but the amount of added uncertainty is expected to lower for every added digital expert. Nevertheless, we must admit, that the uncertainties of our results have to be considered as optimistic estimates, since effects of data error propagation have been ignored.

## 5 Conclusions

We computed probabilistic ensembles of uranium concentration maps covering Norway. To quantitatively account for uncertainty related to the selection of the employed non-linear multiple regression methodology we introduced and applied a digital expert elicitation. In doing so, we employed non-parametric regression algorithms following splitting and continuity-based working hypotheses in parallel and considered their results as equally acceptable votes for possible uranium concentration states. Furthermore, we followed a Monte-Carlo approach to discretely quantify regression model uncertainty emanating from repeated solutions of the regression problem with regard to randomized internal selections of the used algorithms. Albeit our digital expert elicitation widens the uncertainty quantification beyond a probabilistic parameter variation for a single regression model, not all methodological choices and parameter dependencies can be practically studied leaving the probabilistic uranium estimation likely to be overly optimistic. Since no data uncertainty was available for predictor and response variable in the regression problem, our quantified uncertainties exclude the propagation of data uncertainty into the uranium map computation, and must be regarded as adding to potentially overly optimistic character of the achieved results.

Regardless of the employed regression method, we found a strong dominance of mapped geology on the shape of the found regression models. Since several classes of the mapped geology have not been covered by the measured uranium data, our regression models are expected to extrapolate significantly over regions associated to geological classes for which no uranium data were measured when being applied for map computation. Future uranium concentration sampling should strive to cover the pattern in data sets found important for the regression model more evenly. This could help to reduce extrapolation components in the computation of updated uranium maps more efficiently than acquiring more densely sampled data over geological classes covered well already. The employed methodology is of general applicability to regression problems, away from uranium concentration computation, and could be straight forward combined with other Monte-Carlo approaches, i.e., for data uncertainty propagation through the regression. The suggested and employed glyph-based visualization of results enables a loss-free visualization of the probabilistic map results.

## Ethics declarations

Review and/or approval by an ethics committee was not needed for this study because it did not (i) involve chemicals, procedures, or equipment that have any unusual hazards and (ii) involve the use of animal or human subjects.

## Data availability Statement

The data that support the findings of this study are available from the corresponding author upon reasonable request.

## CRediT authorship contribution statement

**Hendrik Paasche:** Writing – review & editing, Writing – original draft, Visualization, Validation, Supervision, Software, Resources, Project administration, Methodology, Investigation, Formal analysis, Data curation, Conceptualization. **Ying Wang:** Writing – review & editing, Visualization, Validation, Software, Resources, Project administration, Methodology, Investigation, Formal analysis, Data curation, Conceptualization. **Vikas Chand Baranwal:** Writing – review & editing, Visualization, Validation, Software, Resources, Project administration, Methodology, Investigation, Formal analysis, Data curation, Conceptualization. **Marco Brönner:** Writing – review & editing, Visualization, Validation, Supervision, Software, Resources, Project administration, Methodology, Investigation, Funding acquisition, Formal analysis, Data curation, Conceptualization.

## Declaration of competing interest

The authors declare that they have no known competing financial interests or personal relationships that could have appeared to influence the work reported in this paper.

## Figures and Tables

**Fig. 13:**
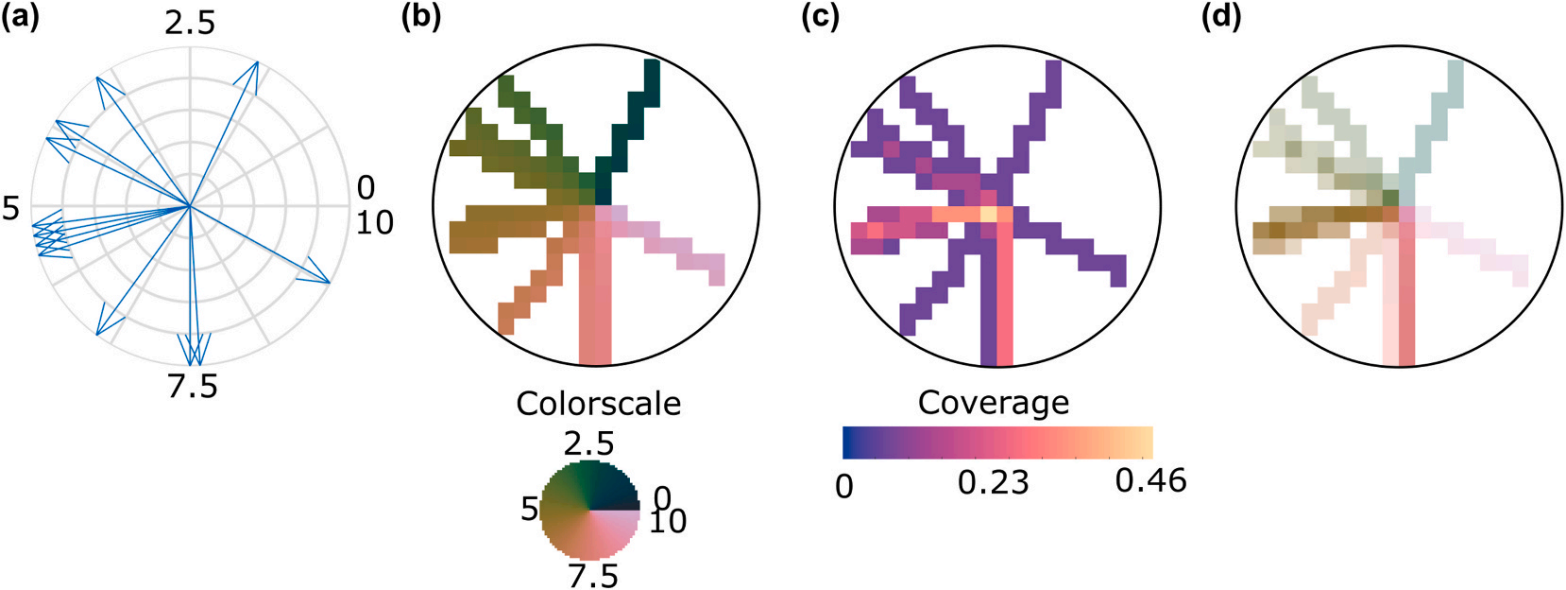
(a) polar plot of a vector with 15 elements. (b) 20x20 pixel glyph showing the same vector as in (a). Pixel coverage of the glyph in (b). Pixels with higher coverage than 1/15 have been used for illustration by more than one element of the vector. (d) the same as in (b), but with coverage information scaled on transparency. Pixels with a coverage above 0.23 are shown with full color saturation. For coverages between 0.23 and 0 the transparency increased linearly to 100 %.

## 11 APPENDIX

The digitated glyph we use here is a simplification of the digitated Bézier glyph [51]. Let us consider the vector **a** = [1.8, 3.5, 4.1, 4.3, 5.2, 5.2, 5.3, 5.4, 5.5, 6.5, 7.5, 7.6, 7.6 7.6, 9.2], which could for example represent uranium concentrations for a map grid node. We can visualize **a** in a polar diagram (Fig. 13a), by mapping its elements on the angle of a circle. Each vector element is depicted by an arrow of equal length originating in the center of the polar diagram. Note, repeated elements in **a** are coincident in the polar diagram. Next, we map the polar diagram in Fig. 13a on a grid with 20 x 20 grid cells (Fig. 13b). The center of the polar diagram is located in the center of the grid. Grid cells intersected by the arrow shaft (ignoring the arrow heads) are activated, all other grid cells remain blank or white. We use a perceptually balanced color scale mapped on a circle. The glyph in Fig. 13b comprises a number of fingers, which partly overlap. Finer grids allow for finer discretization and increasing discriminative power between fingers. Direction and color of the fingers are associated with the vector elements. Since some elements of **a** occur repeatedly, the coverage of the grid cells varies. Since **a** comprises 15 elements, coverage varies in the interval [0, 1] in steps of 1/15. Fig. 13c shows the coverage associated to the glyph shown in Fig. 13b. The digitated glyph in Fig. 13d combines the information of the glyphs in Fig. 13b and c. Color and direction refer to the vector elements, color saturation is related to coverage. We use a color saturation threshold of 0.5, i.e., color saturation between 0.5 times the maximal coverage and zero coverage decreases linearly from full saturation to zero. For grid cells with coverages above 0.5 times the maximal coverage, full color saturation is used. Thus, we emphasize regions of increased data density, i.e., where multiple fingers of the glyph overlap.
